# Physicians’ attitudes towards secondary use of clinical data for biomedical research purposes in Germany. Results of a quantitative survey

**DOI:** 10.1371/journal.pone.0274032

**Published:** 2024-02-13

**Authors:** Anja Köngeter, Christoph Schickhardt, Martin Jungkunz, Katja Mehlis, Eva C. Winkler

**Affiliations:** 1 Section for Translational Medical Ethics, Department of Medical Oncology, National Center for Tumor Diseases, Heidelberg University Hospital, Heidelberg, Germany; 2 Section for Translational Medical Ethics, Department of Medical Oncology, National Center for Tumor Diseases, German Cancer Research Center, Heidelberg, Germany; Universitatsklinikum Schleswig Holstein Campus Lubeck, GERMANY

## Abstract

**Background:**

For biomedical data-driven research purposes, secondary use of clinical data carries great but largely untapped potential. Physicians’ attitudes and their needs towards secondary data use are essential to inform its practical and ethically sound implementation but are currently understudied.

**Objective:**

Therefore, the objectives of the study are to assess physicians’ (i) general attitudes and concerns, (ii) willingness to adapt workflows and to make data available for secondary use, (iii) group-specific conditions toward implementation of secondary use and associated concerns of physician-scientists and purely clinical physicians.

**Methods:**

We developed an online survey based on a literature review and an expert interview study. Physicians in private practice and at two large German university hospitals were surveyed from May 2021 until January 2022.

**Results:**

In total, 446 physicians participated in the survey. 96% [380/397] of all physicians reported a positive attitude towards secondary use; 87% [31/397] are in-principle willing to support secondary use of clinical data along with a small proportion of physicians with fundamental reservations. Secondly, the most important conditions for adapting workflows were funding of additional time and effort for research-adequate documentation (71% [286/390]) and the most important condition for providing patients’ clinical data was reliable protection of patients’ privacy (67% [254/382]). Thirdly, physician-scientists were more likely than purely clinical physicians to request additional funding for research-adequate documentation as a precondition for support (83% vs 69%, *P* = .002) and the privilege to conduct research with their own patients’ clinical data before other researchers are allowed to (43% vs 11%, *P* < .001); while purely clinical physicians more frequently require reliable protection of patient privacy (76% vs 62%, P = .007) and monetary compensation (45% vs 25%, *P* < .001).

**Conclusion:**

Since this study presents high in-principle willingness of physicians to support secondary use along with little general concerns, it seems essential to address physicians’ group-specific conditions toward secondary use in order to gain their support.

## Introduction

Secondary use of clinical data for biomedical research purposes holds promising potential for various types of data-driven research. We define *secondary use* as the collection and reuse of clinical data in data-gathering, non-interventional biomedical research and quality improvement activities [1]. In the past, clinical data collected in healthcare clinical contexts for patient care were not readily available for secondary research, with the exception of clinical data collected in registries for specific diseases. At present, a major data initiative funded by the German Federal Ministry of Education and Research, aims to set up an infrastructure for secondary use of clinical data from health care facilities based on broad consent [2].

In this context, physicians appear to hold a gatekeeper function for clinical data. In Germany, the medical profession refused for years to implement regulations of a data protection compliant standard in medical communication [3]. Consequently, support and political buy-in from the medical profession seems a prerequisite for implementing secondary use [4]. However, physicians‘ attitudes toward secondary use have so far largely been uninvestigated but some studies have focused on related topics. In these studies, data types to be shared are often not clearly characterized and sometimes defined differently preventing direct comparisons. A systematic literature review about researchers’ and health care professionals’ perspectives on data sharing of clinical trial data and health administrative data pinpoints concerns related to the major themes of privacy, user access to data, and potential for misinterpretation of data [5]. Several qualitative studies identified concerns related to secondary use of different kinds of health data also in the populations of general practitioners (GPs) [6–9] and oncologists [10]. It is unclear how pronounced and widespread these concerns are.

No studies exist on the willingness of physicians to implement new workflows for secondary use. We therefore conducted an exploratory interview study in preparation for the present survey: experts of relevant stakeholder groups considered willingness of physicians to provide support for the implementation of secondary use in German hospitals and private practices critical [11]; one GP stated that peers would be reluctant to change their work routine. Regarding monetary incentives to change work routine, two qualitative studies of Australian GPs come to contradictory conclusions [6, 7]. To our knowledge, there are no quantitative studies that examine whether and under what conditions physicians are willing to adjust their work routine for secondary use.

Group distinctions between physician-scientists and purely clinical physicians have not yet been examined. However, two studies indicate disparities between these groups: While Canadian health researchers widely accepted secondary use for research [12] whereas Canadian GPs showed a far lower approval rate [13].

The aim of this study is therefore to assess physicians’ attitudes and group-specific needs regarding secondary use which can inform its practical implementation. To address the delineated research gap, the objectives of the present study are to assess physicians’ (i) general attitudes and concerns, (ii) in-principle willingness to adapt workflows to share data for secondary use, (iii) group-specific conditions to support secondary use in physician-scientists and purely clinical physicians. To our knowledge, we present the first quantitative analysis of physicians’ conditions to support secondary use of clinical data for research purposes and the first study differentiating between physician-scientists’ and purely clinical physicians’ needs.

## Methods

### Survey development

The questionnaire is based on a literature review and the results of an expert interview study [11]. The expert interviews indicated distinct perceptions and expectations of physician-scientists and purely clinical physicians. Therefore, we derived group-specific hypotheses that we operationalized in the form of questionnaire items. The questionnaire was developed and discussed with members of an interdisciplinary research team consisting of social scientists, ethicists, legal scholars, and physicians. To ensure comprehensibility and technical function of the 22 item questionnaire, we pretested the survey by cognitive interviews (n = 6) with physicians with and without experience in generating and using clinical data for research purposes. Based on the results, we adjusted the wording of the specific conditions for physicians’ support to improve comprehensibility. The complete questionnaire is accessible online (https://doi.org/10.11588/data/5JCEVW).

Subsections of the questionnaire include information about i) physician activity and previous experience with secondary use of clinical data, ii) participant’s position on planned research use of clinical data, and iii) possible adjustments to physician activity. To allow participants to develop an informed opinion, the survey included information that the planned secondary use of clinical data from patient care is systematically collected in research databases; the pseudonymized data will be made available for medical research, with the research purpose and research institution not yet determined at the time of data collection. Introductory text also noted the risks and benefits to patients associated with secondary use and mentioned the potential increase in documentary workload for clinicians. The self-administered, anonymous, online survey covered attitudinal questions designed as 5-point Likert scale. Measurement of pronounced research interest was operationalized by asking participants define themselves as physician-scientists; participants who do not define themselves as physician-scientist were referred to as purely clinical physicians. The study obtained ethics approval from the University of Heidelberg’s research ethics committee (reference number S-361/2018). This survey was approved by the data protection officer of Heidelberg University Hospital. These approvals were valid for all data collections.

### Sampling and recruitment

The survey was administered via three data collections: For the first data collection the Cancer Registry of the German federal state of Baden Wuerttemberg sent e-mail invitations to physicians in Baden Wuerttemberg who had reported more than two patients in the registry until April 2021 (full census, N = 3,313). The two most common specializations in the sample frame were GPs (32%), followed by gynecologists (26%). The 2^nd^ and the 3^rd^ data collections complemented the first and was targeted to physicians at university hospitals and thereby a research-oriented environment. For the *2*^*nd*^ *data collection* at the Heidelberg University Hospital (full census, N = 1,686) an e-mail distribution list of all physicians with patient contact was created and was authorized by the university hospital board and the employee council. And for the *3rd data collection* all physicians of the Charité—University Hospital Berlin were invited via email by the Charité-BIH Clinical Study Center (full census, N = 3,870). All e-mail invitations contained a weblink to the anonymous survey.

Individuals who completed the survey were not compensated. Data collections occurred from May 2021 until January 2022 with a duration of 3–4 weeks each. For the 2^nd^ and 3^rd^ data collection, an e-mail reminder was sent out 8 days after the first invitation. For the two surveys at the university hospitals, no overview of the specialties of the physicians contacted was available. Consequently, it was not possible to perform a comparison with the sample for the entire data set. However, the cancer registry provided an overview of the specializations of their sample frame, which allowed us to examine this partial data set. There were no irregularities except for an underrepresentation of general practitioners with only 18.4% instead of 32.1%. Gynecologists, gastroenterologists, and hematologists/oncologists were overrepresented. We hypothesize that primary care physicians may have had less interest in the topic of secondary use or lacked time resources to participate.

### Analysis

Descriptive statistics were used to express categorical variables as counts and percentages. Differences in proportions were assessed for statistical significance (*P<* 0.05) by way of χ2 tests. Significances of group differences in mean values were calculated using the Mann-Whitney U test (two-tailed). All analyses were performed using SPSS IBM version 28.

## Results

### Participant characteristics

Of the 8,615 physicians to whom emails were sent, 446 responded to the survey (response rate: 5%); after excluding participants who answered less than 50% of the items or dropped out before item no. 11, the dataset used for analysis encompassed 397 cases. Of these included physicians, 79% [313/397] worked at a university hospital and 15% [60/397] in a private practice (Table 1). Gender distribution was balanced. 62% [245/397] reported more than 10 years of work experience’.

**Table 1:** Demographics of participants (n = 397).

Characteristics	Values, n (%)
**Affiliation**	
University Hospital / Academic Teaching Hospital	313 (78.64)
Private practice	60 (15.08)
Public community hospitals and for-profit hospitals	20 (5.03)
n/a	4 (1.26)
**Years of Service**	
≤ 10	150 (37.69)
> 10	245 (61.56)
n/a	2 (0.50)
**Medical Speciality (top five)**	
Internal Medicine	88 (22.11)
Surgery	39 (9.80)
Gynecology	39 (9.80)
Anesthesiology	29 (7.29)
Pediatrics and Youth Medicine	25 (5.53)
**Sex**	
Female	180 (45.23)
Male	207 (52.01)
n/a	10 (2.52)
**Identifies as physician-scientist**	
Yes	251 (63.07)
No	125 (31.41)
n/a	21 (5.29)
**Involved in conducting studies using clinical data from patient care within the last 5 years**	
Yes	321 (80.65)
No	72 (18.09)
n/a	4 (1.01)

Overall, 63% [251/397] indicated perceiving themselves as physician-scientist; at university hospitals 84% [243/294] of all physicians considered themselves physician-scientists. 31% [125/397] were defined as purely clinical physicians by indicating that they do not perceive themselves as physician-scientists; 98% [57/58] of all physicians working in private practice were purely clinical physicians. Of all participants, 81% [321/397] reported having contributed to studies using clinical data in the last 5 years.

### General attitudes towards supporting secondary use

**Fig 1:**
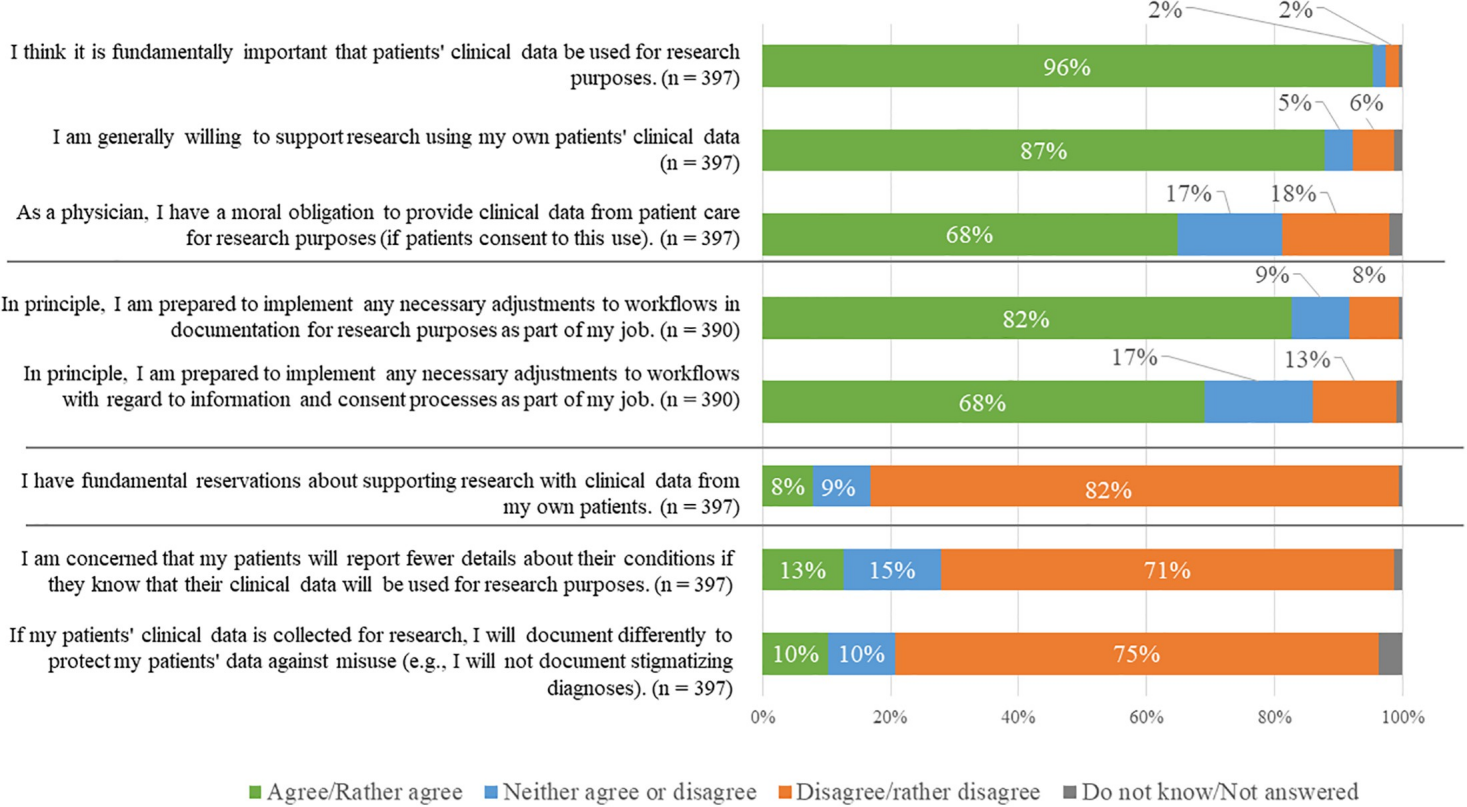
General attitudes and concerns of physicians towards secondary use of clinical data for research purposes and their willingness to support secondary use.

With 96% [380/397] almost all respondents deemed secondary data use for research purposes important and 68% [269/397] hold the view that, as a physician, they have a moral obligation to provide clinical data from patient care for research purposes (Fig 1). Only 8% [31/397] of the participants had fundamental reservations. Yet, 13% [50/397] of respondents were concerned that patients would report fewer details about their illness and 11% [41/397] of physicians would document differently to protect their patients’ sensitive information (e.g. stigmatizing data) against misuse; purely clinical physicians expressed significantly stronger fundamental reservation ($\bar{\text{x}}=2.04$ vs $\bar{\text{x}}=1.50$, *P* < .001), were more concerned that their patients will report fewer details ($\bar{\text{x}}=2.32$ vs $\bar{\text{x}}=1.93$ *P* = .001), and were more inclined to document differently to protect their patients’ data against misuse ($\bar{\text{x}}=2.12$ vs $\bar{\text{x}}=1.73$, *P* = .001) compared to physician-scientists (Table 2). Lack of involvement in research with clinical data within the last 5 years also significantly increased concern that patients would report fewer details (2.38 vs 2.01, P = .011) and tend to document differently (2.35 vs 1.77, P < .001) (data not shown).

**Table 2:** Significance of differences in general attitudes toward secondary use and willingness to support secondary use by self-perception as physician-scientist and purely clinical physician.

Agreement to Statement	Physician scientist, mean (SD)	Purely clinical physician, mean (SD)	*P* value^a^
I think it is fundamentally important that patients’ clinical data be used for research purposes. (n = 397)	4.90 (0.41)	4.53 (0.85)	*<*.*001*
I am generally willing to support research using my own patients’ clinical data. (n = 397)	4.66 (0.81)	4.12 (1.14)	*<*.*001*
As a physician, I have a moral obligation to provide clinical data from patient care for research purposes (if patients consent to this use). (n = 397)	4.12 (1.12)	3.56 (1.27)	*<*.*001*
In principle, I am prepared to implement any necessary adjustments to workflows in documentation for research purposes as part of my job. (n = 390)	4.14 (1.00)	3.56 (1.16)	*<*.*001*
In principle, I am prepared to implement any necessary adjustments to workflows with regard to information and consent processes as part of my job. (n = 390)	4.09 (1.02)	3.48 (1.19)	*<*.*001*
I have fundamental reservations about supporting research with clinical data from my own patients. (n = 397)	1.50 (0.91)	2.04 (1.22)	*<*.*001*
I am concerned that my patients will report fewer details about their conditions if they know that their clinical data will be used for research purposes. (n = 397)	1.93 (1.05)	2.32 (1.16)	.*001*
If my patients’ clinical data is collected for research, I will document differently to protect my patients’ data against misuse (e.g., I will not document stigmatizing diagnoses). (n = 397)	1.73 (1.03)	2.12 (1.21)	.*001*

^a^ Significances of group differences in mean values were calculated using the Mann-Whitney U test; Values in italics are significant at.05 level of significance (two-tailed); 5 Point Likert Scale: Disagree 1; Agree 5.

87% [348/390] of all surveyed physicians were willing to support secondary research use in principle. When being asked about potentially necessary adjustments of the workflow, 71% [286/390] were willing to implement changes for documentation, and 67% [269/390] were willing to inform and consent patients for secondary use. In contrast to purely clinical physicians, physician-scientists were more convinced about the importance of secondary use ($\bar{\text{x}}=4.90$ vs $\bar{\text{x}}=4.53$, *P* < .001), more willing to adjust documentation ($\bar{\text{x}}=4.14$ vs $\bar{\text{x}}=3.56$, *P* < .001), and more willing to obtain informed consent ($\bar{\text{x}}=4.09$ vs $\bar{\text{x}}=3.48$, *P <* .001). Similarly, if the physicians have already been involved in conducting studies with clinical data from patient care in the last 5 years, their willingness to adapt the workflow regarding documentation ($\bar{\text{x}}=4.07$ vs $\bar{\text{x}}=3.45$, P < .001) and regarding informed consent processes ($\bar{\text{x}}=4.01$ vs $\bar{\text{x}}=3.42$, P < .001) was significantly higher than in those without this experience (data not shown).”

### Conditions for adapting workflows

Participants were then asked to indicate what seems most important to them for adapting their workflows in order to support secondary data use. They did so by selecting the three most important conditions out of a list. The most frequently selected conditions were funding of additional person-hours for research-appropriate documentation (77% [302/390]), funding of newly developed and user-friendly software to support research-appropriate documentation (62% [243/390]), and funding of additional person-hours for obtaining informed consent (60% [236/390]) (Table 3). Physicians-scientists and purely clinical physicians differed in only one aspect; funding of additional person-hours for research-adequate documentation was more important to physician-scientists (83% vs 69%, *P* = .002).

**Table 3:** Conditions for adapting workflows for secondary use (documentation and obtain consent); significance of group differences (n = 390).

Conditions to implement adaptations of the workflows^a^	All participants, n (%)	Physician-scientists, n (%)^b^	Purely clinical physicians, n (%)^b^	*P* value^c^
Funding for additional person-hours for research-appropriate documentation	302 (77.44)	205 (82.66)	83 (68.60)	.*002*
Funding of newly developed, user-friendly software to support research-appropriate documentation	243 (62.31)	162 (65.32)	67 (55,37)	.064
Funding for additional person-hours for patient information related to consent	236 (60.51)	153 (61.69)	71 (58.67)	.578
Improving the quality of care across the healthcare system	123 (31.54)	77 (31.04)	38 (31.40)	.945
Improving the quality of care in one’s own practice/department through feedback systems fed by the analysis of clinical data from all integrated practices/departments—including one’s own practice/department	89 (22.82)	50 (20.16)	35 (28.92)	.061
Free access to publications based on routine care clinical data	69 (17.69)	40 (16.12)	26 (21.48)	.207
Certificate in recognition of generating high-quality clinical data for research purposes	23 (5.90)	17 (6.85)	6 (4.95)	.479

^a^ Multiple Answer item; maximum 3 answers.

^b^ Of all participants who answered this item (n = 390), n = 369 indicated whether or not they consider themselves physician-scientists (n = 248) or physicians (n = 121).

^c^ Chi-Squared Test (Pearson); values in italics are significant at.05 level of significance.

When asked about participants’ willingness to spend additional time for research-adequate documentation per patient visit, 53% [165/314] of the participants was willing to document up to additional 2–6 minutes, 25% [79/314] of the participants to document up to 2 minutes, and 22% [70/314] was willing to document > = 6 minutes. A significantly larger proportion of physician-scientists were willing to document > = 6 minutes compared to purely clinical physicians (28% vs 11%, *P* = .005).

63% [249/386] of respondents favoured trained, non-medical staff to inform and consent patients about secondary use; 31% [123/386] felt themselves, as physicians, responsible for this task.

### Conditions for providing patients’ clinical data

All participants were asked about conditions they deem most important in order to make their patients’ clinical data available for secondary use. The most frequently reported conditions were: reliable protection of patients’ privacy (66% [254/382]), extra protection for sensitive data (e.g., genetic data, psychiatric data) (47% [179/382]), and notification about additional/incidental findings relevant to their patients’ health (37% [140/382]) (Table 4).

**Table 4:** Conditions for providing patients’ clinical data available for medical studies and significance of differences in proportions by self-perception as physician-scientist or physician (n = 382).

Conditions to make your patients’ clinical data available^a^	All participants, n (%)	Physician-scientists, n (%)^b^	Purely clinical physicians, n (%)^b^	*P* value^c^
Reliable protection of the privacy of my patients	254 (66.49)	149 (61.57)	91 (75.83)	.*007*
Special protection measures for sensitive data (e.g. genetic data, psychiatric data)	179 (46.85)	108 (44.62)	61 (50.83)	.265
Notification of additional diagnostic findings in my patients that will be identified in the future as a result of planned research use of clinical data (for example, through novel methods of analysis of image data)	140 (36.64)	82 (33.88)	50 (41.66)	.148
Right to initially conduct my own research with my patients’ clinical data	125 (32.72)	104 (42.97)	13 (10.83)	*<*.*001*
Monetary compensation for providing appropriately generated datasets suitable for research purposes	122 (31.93)	60 (24.79)	54 (45.00)	*<*.*001*
Co-authorship of scientific journal articles that use my patients’ data	91 (23.82)	75 (30.99)	10 (8.33)	*<*.*001*
Only with my permission or the permission of my supervisor will researchers receive the clinical data of my patients (without identifying information)	60 (15.70)	45 (18.59)	15 (12.50)	.142
Mention my department/practice in scientific journal articles that use my patients’ data	37 (9.69)	24 (9.92)	13 (10.83)	.787
Mention my name in the acknowledgements of scientific journal articles that use my patients’ data	13 (3.40)	9 (3.80)	3 (2.50)	.542

^a^ Multiple Answer item; maximum 3 answers.

^b^ Of all participants who answered this item (n = 382), n = 369 indicated whether or not they consider themselves physician-scientists (n = 242) or physicians (n = 120).

^c^ Chi-Squared Test (Pearson); values in italics are significant at.05 level of significance.

Patient privacy was significantly more often important to purely clinical physicians than to physician-scientists (76% vs 62%, *P* = .007) as was the monetary compensation for making research-adequate clinical data available (45% vs 25%, *P* < .001). For Physician-scientists the right to first conduct their own research with their patients’ clinical data was more relevant (43% vs 11%, *P* < .001) as was co-authorship in scientific articles based on clinical data of their patients (31% vs 8%, *P* < .001).

Being asked about the acceptance of potential data-users, 40% [159/381] of respondents agreed that all researchers, regardless of their affiliation, should be allowed to use their patients’ clinical data. 46% [174/381] were opposed to making clinical data available for researchers working for companies conducting medical research. 18% [67/381] did not want to provide data for collaborative projects between public research institutions and private companies.

Participants were asked about data ownership. They were divided on whether data can be owned (51%) or not (49%). Among those who held that data can be owned, 54% considered patients as data owners, and 40% believed that the practices and hospitals where data are collected or the physicians own the data, and only 6% thought the data belong to everyone who can use the data to add value to medical care. Purely clinical physicians were significantly more likely to think that data belong to patients (66% vs 48%, P = .024). Interestingly, the view that data can be owned was positively associated (eta = .191 P < .001) with the willingness to provide patients’ clinical data.

#### Concerns about provision of patient data

Participants were asked about their specific concerns with respect to the provision of their patients’ clinical data for secondary use. These concerns differed significantly between the two groups (Table 5). Compared to physician-scientists, purely clinical physicians were significantly more likely to have concerns about misuse of their patients’ clinical data through unauthorized access to datasets by third parties (75% vs 53%, *P* < .001), failure to protect their patients’ privacy (62% vs 36%, *P* < .001), and discrimination based on clinical data against their patients (27% vs 15%, *P* = .009). Involvement in conducting studies with patients’ clinical data in the past 5 years had no significant influence on concerns about misuse of patient clinical data. Purely clinical physicians were also more likely to have concerns about increased liability risk (e.g., research uncovers a misdiagnosis in their own practice/department) (36% vs 26%, *P* = .044), and a loss of patients’ trust in the physician-patient relationship (21% vs 12%, *P* = .019). The second most common concern of physician-scientists (after concerns about privacy of their patients) was insufficient data quality that can result in inaccurate findings (62% vs 42%, *P* < .001), and that other researchers conduct research using their patients’ clinical data before they do (43% vs 13%, *P* < .001).

**Table 5:** Concerns about providing patients’ clinical data for secondary use and significance of group differences (n = 382).

Concerns about making patients’ clinical data available^a^	all participants, n (%)	physician-scientists, n (%)^b^	Purely clinical physicians, n (%)^b^	*P* value^c^
Misuse of data through unauthorized access to data records	229 (59.94)	127 (52.47)	90 (75.00)	*<*.*001*
Insufficient data quality, which can cause studies to produce erroneous results	215 (56.28)	151 (62.39)	50 (41.66)	*<*.*001*
Failure to adequately protect the privacy of my patients	174 (45.54.	87 (35.95)	74 (61.66)	*<*.*001*
Other researchers conduct research using the clinical data from my practice/department before I do	124 (32.46)	104 (42.97)	15 (12.50)	*<*.*001*
Novel liability issues/increased liability risk (e.g., research uncovers misdiagnosis in own practice/department)	115 (30.10)	62 (25.61)	43 (35.83)	.*044*
New technological developments with new possibilities for re-identification carry the potential for harm to my patients	89 (23.29)	51 (21.07)	32 (26.66)	.233
Discrimination against my patients on the basis of their clinical data, e.g. in the case of re-identification of individual patients or on the basis of belonging to a group with a certain disease or disposition	74 (19.37)	37 (15.28)	32 (26.66)	.*009*
Performance comparisons with other practices/departments at hospitals conducted using clinical data from external agencies	71 (18.58)	47 (19.42)	21 (17.50)	.659
Loss of trust in the doctor-patient relationship on the part of the patients	61 (15.96)	28 (11.57)	25 (20.83)	.*019*
Future studies pursue research purposes that harm my patients	31 (8.11)	16 (6.61)	11 (9.16)	.384

^a^ Multiple Answer item.

^b^ Of all participants who answered this item (n = 382), n = 369 indicated whether or not they consider themselves physician-scientists (n = 242) or physicians (n = 120).

^c^ Chi-Squared Test (Pearson); values in italics are significant at.05 level of significance.

## Discussion

### Main findings

Information on whether and under what preconditions physicians are willing to make their patients’ clinical data available for research is vital for practical and ethically sound implementation of secondary use. We report results of a survey among 397 physicians working in two university medical centres and in private practice on their general attitudes, concerns, in-principle willingness and conditions for enabling support of secondary use of clinical data for research purposes. To our knowledge, we present the first quantitative analysis of physicians’ conditions to support secondary use of clinical data for research purposes.

Firstly, we found a highly positive general attitude of physicians towards secondary use along with little fundamental reservations. Secondly, physicians showed widespread in-principle willingness to support secondary use; most important conditions for practical implementation were reliable protection of patients’ privacy as well as funding of additional person-hours for documentation and consenting patients. Third, group specific differences were prevalent: Physician-scientists were more likely to be concerned about data quality, required additional funding for research-adequate documentation and the right to first conduct research with their patients’ clinical data; in contrast, purely clinical physicians were more prone to be concerned about patient privacy and the physician-patient relationships, and to require, hence, reliable patient privacy as well as monetary compensation.

### High in-principle willingness for supporting secondary use

Despite methodological and thematic differences that limit comparability, we aim to embed our findings carefully within related studies. Among all 397 surveyed physicians, a very high proportion of physicians were of the view that secondary use is important (96%) and were in-principle willing to support secondary use (87%). This corresponds with a previous survey in a small sample in Canadian health researchers presenting the very strong general acceptance (96%) of using “citizens’ health data for research” [12]. Our study further found a majority of physicians viewing the support of secondary use as a moral duty of peer physicians (68%). This view resonates with the results of a study we have previously conducted in which cancer patients attributed an obligation to their physicians to support secondary use (91%) [14]. An ethical discussion of such a duty to support secondary use seems essential in order to resolve the tension of conflicting duties.

The finding that only few physicians expressed fundamental reservations about secondary use (8%) helps to classify, at least for the German context, the presumption expressed in qualitative studies about severe concerns [6, 7, 13].

This study showed that making clinical data available for researchers who work for companies and conduct medical research was acceptable for the majority of physicians (40%). This aspect was previously considered problematic in qualitative studies among physicians [7, 10, 15]. Acceptance rate in our study was considerably higher than in a small sample of GPs conducted in Canada being asked for use of electronic health record data for research by pharmaceutical industry (9%; n = 46) [13]. This discrepancy should be examined in a more nuanced way with a focus on types of data use and consideration of wordings such as ’pharmaceutical industry’ which could carry negative connotations. Consistent with findings of a qualitative study with GPs conducted in the UK [8], physicians were willing to support secondary use in case of public-private-partnerships which could be an alternative to purely private use.

### Most important conditions for practical implementation

#### Adjustment of workflows

About three-quarters of physicians were in principle willing to adjust their workflows to support secondary use (76%). Physicians were most interested in keeping expenditures in terms of time, personnel and money to a minimum, a finding that is consistent with results of a Canadian qualitative study in GPs [7]. Studies report that physicians are already—even without documentation for research purposes–increasingly dissatisfied with the time spent on documenting in electronic medical records [16, 17] with one quantitative study even suggesting electronic health record usage is related to burnout [18]. Hence, physicians will likely be sensitive towards spending extra time that could reduce contact time with their patients. While physicians in this study indicated that additional documentation time of 5 minutes on average per patient visit would be acceptable, they simultaneously requested extra funding for personnel for research-adequate documentation of data (77%). Software solutions might be apt to reduce this burden, if they decrease time for documentation significantly [19], and cover a range of functional tasks [20]. During the development of new software and workflows, healthcare personnel and hospital management should be directly involved at an early stage [6]. To provide direct benefits and incentives for medical care, feedback systems can improve internal quality and recognition of efforts and, hence, contribute to a learning healthcare system by strengthening the link between care and research.

The majority of physicians agreed that non-medical staff should consent patients for secondary use (62%). We suppose that physicians clearly distinguish consent to interventional clinical trials from consent to *non-interventional* secondary research use. It seems worthwhile to consider obtaining informed consent for secondary use by trained, non-medical personnel.

#### Provision of patient data

Compared to physician-scientists, purely clinical physicians were less inclined to make their patients’ clinical data available for research purposes ($\bar{\text{x}}=4,66$ vs $\bar{\text{x}}=4,12$, *P* < .001). This finding resonates with a survey among GPs from Canada with moderate acceptance rate of sharing patient data with university researchers (60%) [12]. Also, this group was more likely to see patients as the owner of the data while, overall, the positions on whether data can be owned at all was quite divided. This reflects the controversial ethical debate about the concept of ownership with regard to health data where convincing arguments would rather support ownership in a sense of control and engagement than in the sense of property [21].

The most important condition to make clinical data available for secondary use was the reliable protection of patient privacy (67%) being consistent with existing literature indicating that physicians feel responsible for patient privacy and view themselves as data custodians [7, 22]. To build trust in secondary use, implementation of reliable data security and data protection seems essential as well as informing physicians about data protection measures—and also about data leaks if they occurred.

### Addressing physician-scientists’ research interests

We found that physician-scientists and purely clinical physicians differed systematically. Physician-scientists’ research experiences and interests might be the reason for their significantly stronger concerns about insufficient data quality for research purposes. This finding is in line with a mixed-method study among health researchers [23]. To ensure physician-scientists’ trust in research-adequate data quality, implementing appropriate measures and resources for high quality documentation should receive high priority. Physician-scientists were also more likely than purely clinical physicians to demand additional funding for research-adequate documentation. In the context of the healthcare personnel’s double burden of care and research, the study by Orton et al. calls for supporting physician-scientists to conduct research activities alongside patient care [24]. In addition to such support, physicians should simultaneously be made aware that their documentation practices have direct consequences for the scientific usability of clinical data and for the quality of research results.

Compared to purely clinical physicians, physician-scientists emphasised significantly more often the privilege to conduct research with patient data prior to other researchers which is in line with a systematic literature review demonstrating that health researchers want to exert some control over data they had collected [5]. Potential rights of–time-limited—exclusive use might facilitate the implementation of secondary use, but need to be weighed against the argument of maximizing utility of data generated in a publicly funded healthcare system. Other ways of recognition might be considered such as co-authorship of physicians who collected clinical data.

### Addressing purely clinical physicians’ interests

Purely clinical physicians were significantly more often concerned than physician-scientists about their patients loosing trust in the physician-patient relationship if data are made available for secondary use. Such concerns have not been reported so far. Purely clinical physicians were also more likely to be concerned about protection of their patients’ privacy and placed reliable privacy protection as the most important condition to support secondary use. They even reported to protect their patients’ data by documenting differently. According to the literature, trust in data users seems to be a vital facilitator for secondary use [7, 23, 25] as well as a trustworthy governance structure and oversight bodies, e.g. use and access committees [15, 26]. For this group, it seems vital to build trust in data infrastructure and governance of secondary use by ensuring and communicating patient privacy protection. Similarly, physicians who had not been involved in conducting studies with clinical data studies within the last 5 years showed increased concerns and a lower willingness to adapt workflows which suggests greater involvement in medical studies of all physicians during their training and their career may lower concerns and increase willingness to adapt workflows.

An important incentive for purely clinical physicians was monetary compensation of expenses for secondary use. Since almost all purely clinical physicians work in private practices, this finding might reflect their economic situation as mostly self-employed entrepreneurs. Since purely clinical physicians typically do not plan to use and directly benefit from the preparation and provision of data, fair compensation schemes seem imperative for this group [7].

## Limitations

The low response rate (5%) reflects difficulties to motivate physicians to participate in studies have been recognized [27] with reasons for non-participation such as survey fatigue and minimal time resources. Also, the subject of the present study does not directly address topics relevant to patient care which may have further reduced interest in participation. Given the low response rate, self-selection bias cannot be ruled out. The high proportion of physicians working at university hospitals and an underrepresentation of general practitioners might lead to a study population with increased research interest and research familiarity, possibly overstating positive attitude towards secondary use. In Germany, however, the collection of clinical data is to be introduced in university hospitals first, so that representation of this group seems appropriate. We used a self-developed questionnaire without validated measurement instruments, yet tested by cognitive interviews. The sample is not representative of the German medical profession, yet our results may provide indications of relevant needs and concerns of physicians in Germany.

We assumed that organizational background exerts relevant influence on physicians’ attitudes toward secondary use as physicians working in private practice potentially feel more in charge for data protection standards of their practice than physicians working in a hospital. The distinction between physicians working in hospitals and in private practice needs further assessment in a larger dataset to inform implementation of secondary use in different organisational settings.

## Conclusion

This first quantitative study on the perspective on secondary health data use of physicians in an research prone environment compared to those in private practice should inform further studies and the setup of infrastructures for secondary use of clinical data in Germany and possibly beyond. We found high in-principle willingness of physicians to support secondary use and low general concerns. High in-principle willingness and little concerns indicate the importance of considering physicians’ demands and conditions in order to foster secondary use: most important conditions were protection of patient privacy and manageable expenses in terms of time, personnel and money. If extra expenses occur, the provision of funding to compensate for them is expected such as medical documentation specialists, non-medical staff obtaining consent, and user-centred documentation software—in order to not further reduce contact time with patients. Adaptation of workflows for research-adequate documentation and consenting patients should be pilot-tested in participatory (research) formats in order to prevent disruption of complex clinical processes.

Our results demonstrated group-specific differences. *Physician-scientists’* answers mirrored the rationales of the scientific system with concerns about research-adequate data quality, requesting incentives such as the privilege of first conducting research and funding for research-adequate documentation. Building trust in data repositories and its users seems essential for physician-scientists’ support and readiness to conduct research with clinical data themselves. *Purely clinical physicians* were concerned about patients’ privacy and about a possibly worsening physician-patient relationship. Their most important condition for support of secondary use was the protection of patient privacy but also monetary compensation which can be attributed to the often self-employed work in private practices performed by this group. Besides establishing monetary compensation schemes, for purely clinical physicians, relevant conditions to support secondary use include ensuring and communicating patient privacy protection accompanied by a trustworthy data governance structure that enables transparent data use.
